# Regeneration of a Rabbit Segmental Defect with a New Bone Therapy: Autologous Blood Coagulum with Bone Morphogenetic Protein 6 and Synthetic Ceramics

**DOI:** 10.34133/bmr.0140

**Published:** 2025-02-05

**Authors:** Nikola Stokovic, Natalia Ivanjko, Ana Javor, Marko Pecin, Katarina Muzina, Zeljka Magdalena Stepanic, Hrvoje Capak, Zoran Vrbanac, Drazen Maticic, Slobodan Vukicevic

**Affiliations:** ^1^ Laboratory for Mineralized Tissues, School of Medicine, University of Zagreb, Zagreb, Croatia.; ^2^ Center of Excellence for Reproductive and Regenerative Medicine, Zagreb, Croatia.; ^3^ Department of Radiology, Ultrasound Diagnostics, and Physical Therapy, Faculty of Veterinary Medicine, University of Zagreb, Zagreb, Croatia.; ^4^ Clinics for Surgery, Orthopedics, and Ophthalmology, Faculty of Veterinary Medicine, University of Zagreb, Zagreb, Croatia.; ^5^ Department of Inorganic Chemical Technology and Non-metals, Faculty of Chemical Engineering and Technology, University of Zagreb, Zagreb, Croatia.

## Abstract

Segmental defects of long bones are among the most challenging and debilitating conditions in clinical medicine. Osteogrow-C is a novel osteoinductive device composed of recombinant human bone morphogenetic protein 6 (rhBMP6) delivered within autologous blood coagulum (ABC) with calcium phosphate ceramics that was proven efficacious in preclinical models of spinal fusion. This study aimed to evaluate the efficacy of Osteogrow-C in comparison to that of other osteoinductive therapies in a clinically relevant segmental defect model in rabbits. Segmental defects (15 mm) of rabbit ulna were treated with Osteogrow-C containing different synthetic ceramic particles (tricalcium phosphate [TCP] and TCP/hydroxyapatite 40%/60%), Osteogrow (rhBMP6/ABC), Infuse (rhBMP2/absorbable collagen sponge), and control implants without bone morphogenetic proteins. Defect healing was evaluated by in vivo x-ray scans 4, 8, and 17 weeks after the surgery, and animals were killed after 17 weeks for further radiographical and histological assessment. Evaluation of x-ray images, micro-computed tomography, and histological sections revealed that both Osteogrow-C formulations as well as Osteogrow and Infuse promoted healing of the ulnar segmental defect. However, radiographic scores were higher in animals treated with Osteogrow-C than those for the other used therapies. Moreover, evaluation of in vivo x-ray scans revealed that Osteogrow-C with TCP ceramics induced the most rapid defect bridging. On the other hand, control implants (ABC/TCP and ABC/biphasic calcium phosphate) promoted limited osteogenesis without defect bridging. The findings of this study suggest that Osteogrow-C is a promising safe therapeutic solution for the treatment of large bone defects, providing relief to millions of patients suffering from this debilitating condition.

## Introduction

Segmental defects of long bones are defined as the absence of bone in a length longer than one and a half of the bone diameter [1]. Such defects might result from extensive resection of bone tumors, severe limb injury, osteomyelitis, or congenital malformation [2]. Although relatively rare, segmental defects are debilitating conditions due to a substantial decrease in a patient’s life quality with the possibility of limb amputation [2]. Treatment of large segmental defects is among the most challenging conditions in orthopedics because they cannot heal by endogenous self-repair mechanisms and therefore require a surgical intervention [3,4]. Currently, the gold standard for treating large segmental defects is autologous bone graft (ABG) harvested from the iliac crest that possesses osteogenic, osteoinductive, and osteoconductive properties [1,5]. However, the amount of available iliac crest bone is limited and harvesting eventually leads to deformity and scarring at the donor site, increased blood loss, and risk for infection [6]. Bone morphogenetic proteins (BMPs) are powerful growth factors, some of which can induce osteogenesis, forming the basis for novel therapies potentially replacing ABGs in treating segmental defects [7]. Osteoinductive BMPs require a biocompatible and biodegradable carrier that has affinity for BMPs, enables sustained release, and has sufficient porosity for cell infiltration and neoangiogenesis to form bone [8–10]. A large number of preclinical studies were focused on the evaluation of different natural and synthetic polymers and inorganic materials as potential BMP carriers in segmental defect models [11–22]. However, none of these therapies were approved for clinical use in the treatment of large segmental defects. Currently, the only clinically approved BMP-based therapy is the Infuse bone graft (Medtronic, MN, USA) composed of recombinant human bone morphogenetic protein 2 (rhBMP2) delivered on absorbable collagen sponge (ACS). Infuse is approved for application in the anterior lumbar interbody fusion procedure, for the treatment of acute tibial fracture and sinus floor lifting.

We have developed Osteogrow, a novel ABG substitute composed of recombinant human bone morphogenetic protein 6 (rhBMP6) and autologous blood coagulum (ABC) as a BMP carrier [7,23]. The safety and efficacy of Osteogrow were evaluated in preclinical models in rats, rabbits, and sheep and phase I/II clinical trials in patients with distal radial fracture and patients undergoing high tibial osteotomy [10,22,24–26]. However, preclinical studies have revealed that in certain indications where compressive forces are present such as posterolateral spinal fusions (PLFs) and management of large segmental defects, Osteogrow implants should be supplemented with compressive resistant matrix (CRM) [27]. At first, we evaluated Osteogrow with allograft bone particles (Osteogrow-A) in preclinical spinal fusion models (rabbit and sheep PLF models) and patients undergoing posterior lumbar interbody fusion (EudraCT number: 2017-000860-14) [24,27]. Since allografts are associated with several disadvantages, including possible immune rejection, regulatory issues, and disease transmission risk [1,28], we substituted allografts with synthetic calcium phosphate ceramics in the form of tricalcium phosphate (TCP), hydroxyapatite (HA), or biphasic calcium phosphate (BCP) ceramics containing TCP and HA in different ratios. We conducted an extensive preclinical evaluation of Osteogrow-C (Osteogrow containing synthetic ceramics) properties and demonstrated its superior efficacy compared to that of Osteogrow-A [29–35].

Recently, a 2-year-old dog with a large, 3-cm-long multisegmental gunshot defect of the humerus was treated surgically along with the insertion of an external fixator at the Clinics for Surgery, Orthopedics, and Ophthalmology at the University of Zagreb Faculty of Veterinary Medicine [36]. However, 3 months following surgery, there was a complete lack of bone formation, and to avoid limb amputation, Osteogrow-C containing TCP particles was applied in the second surgery. Osteogrow-C induced rapid bridging of segmental defects at 3 months following implantation, and the limb function was restored 1 year following surgery [36].

Successful healing of the aforementioned defect in a dog prompted us to conduct this study to evaluate Osteogrow-C in a rabbit ulnar segmental defect model. Furthermore, we compared the outcome following implantation of Osteogrow-C implants containing 2 different chemical compositions of ceramics (TCP and BCP ceramics containing TCP and HA in a 40/60 ratio) with those of Infuse bone graft (rhBMP2/ACS), Osteogrow (rhBMP6/ABC), and implants containing only ceramic particles in ABC.

## Materials and Methods

### Experimental design

We evaluated the healing of a ulnar segmental defect in rabbits following implantation of Osteogrow-C with synthetic ceramic particles that differed in chemical composition (TCP and TCP/HA 40%/60%), Osteogrow (rhBMP6 in ABC), Infuse bone graft (rhBMP2 on ACS), and control implants without BMPs.

**Fig. 1.**
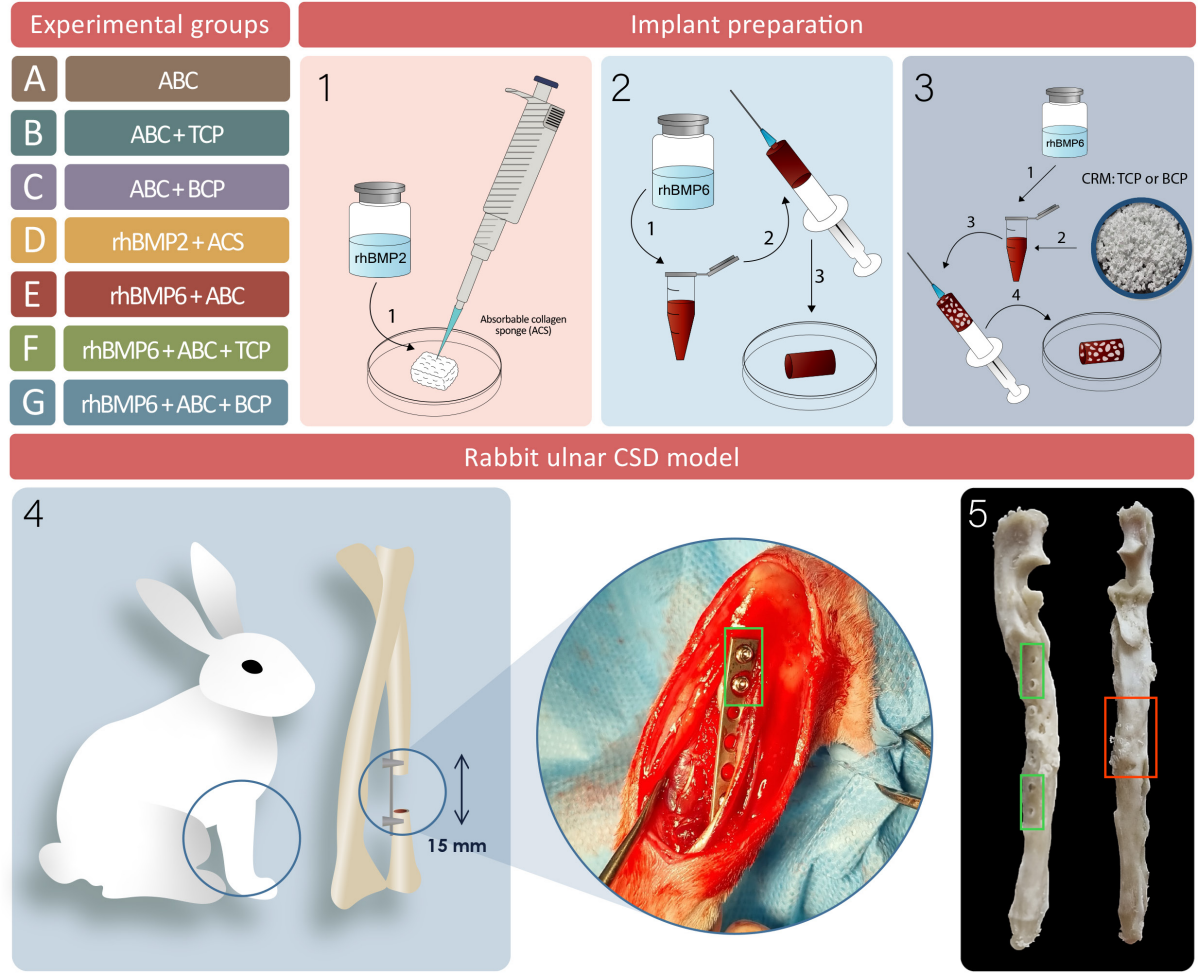
Experimental groups, implant preparation, surgical technique, and outcome for the macerated specimen of a rabbit forearm showing a regenerated ulnar segmental defect. Implant preparation of (1) Infuse (recombinant human bone morphogenetic protein 2 [rhBMP2]/absorbable collagen sponge [ACS]), (2) Osteogrow (recombinant human bone morphogenetic protein 6 [rhBMP6]/autologous blood coagulum [ABC]), and (3) Osteogrow-C containing tricalcium phosphate (TCP) or biphasic calcium phosphate (BCP). (4) Schematic preview of a rabbit critical size defect (CSD) model with a defect of 15 mm stabilized with a metal plate. (5) Macerated specimen of the rabbit forearm and regenerated ulnar defect using Osteogrow-C containing BCP (orange rectangle) and screw holes from the removed plate (green rectangle). CRM, compressive resistant matrix.

Experimental animals were assigned to the following 7 experimental groups: (A) ABC, (B) ABC + TCP ceramics (particle size 1,000 to 1,700 μm), (C) ABC + BCP ceramics (BCP with TCP/HA ratio of 40%/60%, 1,000 to 1,700 μm), (D) rhBMP2/ACS, (E) rhBMP6/ABC, (F) rhBMP6/ABC + TCP (particle size 1,000 to 1,700 μm), and (G) rhBMP6/ABC + BCP ceramics (TCP/HA ratio 40%/60%, particle size 1,000 to 1,700 μm) (Fig. 1).

The applied dose of rhBMP6 was 100 μg per implant, while the amount of ceramics in implants containing CRM was 200 mg. The number of animals treated with BMP-based osteoinductive implants (groups D to G) was 4 to 5. The sample size was determined based on literature data and recommendations for conducting animal experiments [11]. On the other hand, the number of animals treated with control implants without BMP (groups A to C) was minimal (1 to 2) according to the request of the institutional ethics committees that reviewed this study to minimize the number of experimental rabbits in control groups with therapies previously shown to be ineffective in new bone induction.

Healing of the segmental defect was assessed by in vivo x-ray scans 4, 8, and 17 weeks following the surgical procedure. Experimental animals were killed after 17 weeks for further assessment of segmental defect bridging by ex vivo x-ray scans, micro-computed tomography (microCT), and histological analyses.

### Characterization of synthetic ceramic particles

Prior to conducting animal experiments, synthetic ceramic particles (CaP Biomaterials, WI, USA) were characterized by scanning electron microscopy (SEM), energy-dispersive x-ray spectroscopy (EDS), Fourier transform infrared spectroscopy (FTIR), and x-ray diffraction (XRD) analysis. The morphology and particle size were determined using SEM. Sample preparation consisted of fixing the samples onto aluminum sample holders with double-sided carbon conductive tape and applying a layer of gold and palladium using a Quorum SC 7620 sputter coater. SEM analysis was conducted using a Tescan Vega 3 EasyProbe scanning electron microscope (Tescan, Czech Republic) operating at an accelerating voltage of 10 kV. The microscope was equipped with a Bruker B-Quantax EDS detector, which facilitated the determination of the elemental composition of the samples. FTIR spectra were obtained with a Bruker Vertex 70 FTIR spectrometer (Bruker Optics, Germany) operating in attenuated total reflectance mode with a spectral resolution of 2 cm^−1^ and an average of 32 scans. XRD analysis was performed with Shimadzu XRD 6000 apparatus (Shimadzu, Japan) with Cu Kα irradiation, an accelerating voltage of 30 kV, and a current of 30 mA, operating in step scan mode with 0.02° 2*θ* steps and 0.6-s retention time.

**Fig. 2.**
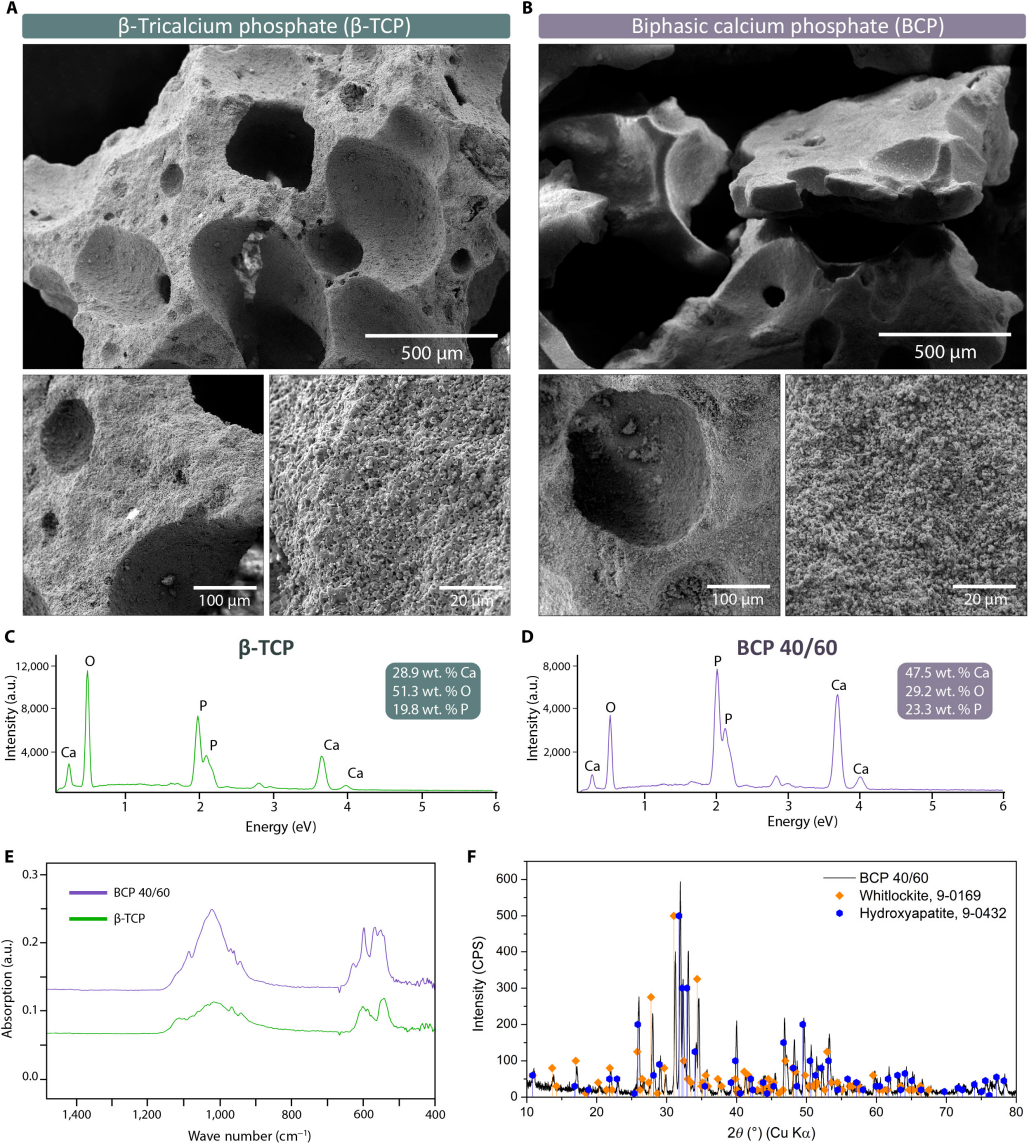
Characterization of synthetic ceramic particles. Scanning electron microscopy (SEM) micrographs of (A) β-TCP and (B) BCP 60/40 ceramic particles showing varying particle sizes (size range from 1,000 to 1,700 μm), irregular shapes, and porous morphology. Energy-dispersive x-ray spectroscopy (EDS) spectra of (C) β-TCP particles and (D) BCP 60/40 particles, (E) Fourier transform infrared spectroscopy (FTIR) spectra (fingerprint region) of β-TCP (green) and BCP particles (purple), and (F) x-ray diffraction (XRD) pattern of BCP particles.

SEM analysis (Fig. 2A and B) revealed that the synthetic ceramic samples comprised varying particle sizes, irregular shapes, and a porous morphology, characteristics advantageous for the intended application. The average pore size for TCP ceramics was 168 μm, while that for BCP ceramics was 148 μm. According to the results of the EDS analysis (Fig. 2C and D), there was a visible difference in the proportions of the elements present. Both samples consisted of calcium, phosphorus, and oxygen, while the differences in element ratios were the consequence of the different phase compositions of the samples. The unassigned peaks in the EDS spectra belonged to gold and palladium from the applied conductive layer. Additionally, the low-intensity phosphorus peak at 2.1 eV overlapped with an intensive gold peak; therefore, the calculated element ratios can only be considered approximate.

The FTIR spectra (Fig. 2E) of the studied ceramic particles were similar, which is to be expected considering their similar chemical compositions. Bands for individual PO_4_^3−^ vibrational modes were in the same region for both samples, while the biphasic sample BCP 40/60 had additional bands for all PO_4_^3−^ vibrational modes and an additional band corresponding to the OH– group, resulting from the presence of the HA phase in the sample.

XRD analysis was performed solely on the biphasic ceramic sample to confirm its phase composition. The obtained XRD pattern (Fig. 2F) consisted of peaks corresponding to synthetic HA, Ca_5_(PO_4_)^3^(OH) (ICDD PDF-2 9-0432), and synthetic whitlockite, Ca_3_(PO_4_)^2^ (ICDD PDF-2 9-0169), which meets the manufacturer’s specifications.

### Experimental animals

The study was conducted in 24 New Zealand White rabbits (lat. *Oryctolagus cuniculus*, 20 weeks old, male, a body weight of 3 to 5 kg). Experimental animals were housed in the registered animal facility of the Laboratory for Mineralized Tissues (HR-POK-001) at the University of Zagreb School of Medicine providing conventional laboratory conditions (temperature 18 to 24 °C, relative humidity 50% to 70%, illumination 12 h/d, and noise level 60 dB). Approval for the studies was given by the Directorate for Veterinary and Food Safety, Ministry of Agriculture, Republic of Croatia, following evaluation of the Ethics Committee at the University of Zagreb School of Medicine and the National Ethics Committee (EP 332/2021). The ethical principles of the study ensured compliance with European Directive 2010/63/EU, the Law on Amendments to the Animal Protection Act (Official Gazette 37/13), the Animal Protection Act (Official Gazette 102/17), the ordinance on the protection of animals used for scientific purposes (Official Gazette 55/13), Animal Research: Reporting of In Vivo Experiments guidelines, and Federation of European Laboratory Animal Science Associations recommendations.

### Implant preparation

In control groups (A, B, and C), autologous blood was withdrawn from the ear marginal vein and left to coagulate immediately (group A) or after mixing with 200 mg of TCP (group B) or BCP (group C) ceramic particles. Implants composed of rhBMP2/ACS (group D) were prepared by uniform application of rhBMP2 solution (100 μg; Medtronic BioPharma B.V., The Netherlands) to ACS (Integra LifeSciences Corporation, NJ, USA) with dimensions of 12.7 mm × 25.4 mm × 7 mm (Fig. 1). Osteogrow-C and Osteogrow implants were prepared according to the following procedure (Fig. 1): lyophilized rhBMP6 (Genera Research, Zagreb, Croatia) was dissolved in water and added to autologous blood withdrawn from the ear marginal vein (100 μg of rhBMP6 in 1 ml of blood). Autologous blood containing rhBMP6 was left to coagulate immediately (group E) or following the addition of TCP (group F) or BCP (group G) ceramics (200 mg per implant) according to the experimental design (Fig. 1). The synthetic calcium phosphate ceramics used in this study were produced by CaP Biomaterials (WI, USA) and were evaluated in previous Osteogrow-C preclinical trials [29–32]. All implants were implanted within 1 h following the preparation and blood coagulation [22].

### Surgical procedure

The surgical procedure was conducted at the Clinics for Surgery, Orthopedics, and Ophthalmology (University of Zagreb Faculty of Veterinary Medicine), according to established protocols [22]. In brief, at the beginning of the procedure, animals were premedicated with ketamine (50 mg/kg), acepromazine (1 mg/kg), and xylazine (5 mg/kg). Anesthesia was maintained during surgery with a mixture of isoflurane (1% to 1.5%) and oxygen. The surgical procedure started with a skin incision on the lateral part of the leg that was followed by a dissection of the musculature overlying the ulna. A large, 15-mm segmental defect of the ulna was created in the ulna and filled with an implant according to the experimental design of the study (Fig. 1). To ensure limb mechanical stability, the radius was left intact while 2 portions of the ulna were fixed using a fixator.

### Radiological monitoring

Healing of the ulnar segmental defect was monitored by in vivo x-ray images obtained 4, 8, and 17 weeks following surgery at the Department of Radiology, Ultrasound Diagnostics, and Physical Therapy (University of Zagreb Faculty of Veterinary Medicine). Animals were sedated before radiological examination with a combination of intramuscular administration of ketamine (Narketan, Vetoquinol, Switzerland) at a dose of 30 mg/kg and xylazine (Xylapan, Vetoquinol, Switzerland) at a dose of 3.5 mg/kg. All experimental animals were terminated 17 weeks after surgery by intrapulmonary administration of T61 (1 ml/kg) following premedication with ketamine and xylazine. Following euthanasia, the rabbit ulna and radius were harvested, and final x-ray scans were obtained. Next, metal plates were removed from all specimens to improve the quality of the microCT scans, as the presence of metal causes artifacts that interfere with the accuracy of microCT analyses.

### MicroCT analyses

At the end of the follow-up period, the obtained specimens were scanned by a 1076 SkyScan μCT device (Bruker, Belgium) according to a previously described procedure to analyze bone regeneration and osseointegration [30–32]. In brief, the scanning resolution was 18 μm; the frame averaging, 2; and the rotation step, 0.5°. Obtained images were reconstructed using the NRecon software (Bruker, Belgium) and analyzed using the CTAn software (Bruker, Belgium). MicroCT scans were used to analyze bone formation, defect bridging, and the resorption rate of ceramic particles. Healing of the segmental defects was analyzed using the same radiographic grading score system as for x-ray images [37]. Moreover, microCT analyses revealed the bone volume, cortical thickness, and trabecular parameters (trabecular thickness, trabecular number, and trabecular separation) of the induced bone.

### Bone maceration

Bone maceration of the selected specimen was conducted according to our standard procedure as previously described [32]. Shortly, the majority of residual muscles and connective tissue were manually removed. Next, the specimen was placed in water with the addition of a detergent to remove fat and heated at a constant temperature for several days.

### Histology

Harvested bones were placed in 10% formalin for 10 d to achieve fixation and processed decalcified or undecalcified as previously described [30–32]. In brief, the majority of specimens were decalcinated using 14% EDTA in 4% formalin solution for 20 d and subsequently paraffin-embedded. Selected samples were processed undecalcified; they were dehydrated using graded solutions of ethyl alcohol and polymerized into hardened acrylic resin blocks. Embedded specimens were cut to obtain 5-μm sections that were mounted on gelatin-coated glass slides and finally stained by Goldner, hematoxylin-eosin, or Von Kossa stain.

### Grading scales and data management

The grading system used in this study was the radiographic scale proposed by Cook et al. [37]. Radiographic improvement is stratified across 6 grades (1 to 6), while 0 indicates no change from immediate postoperative appearance. This scale was used by 3 independent evaluators (N.S., N.I., and A.J.) with expertise in bone imaging to evaluate the bone healing of ulnar segmental defects treated with various experimental groups. Evaluators rated the healing of segmental defects of in vivo x-ray images taken on the 4th, 8th, and 17th weeks after surgical procedure and implantation. Furthermore, the same grading scale was used for the evaluation of the ex vivo microCT sections of samples in the sagittal plane. Grading was conducted in a blinded and independent fashion. To assess the interrater reliability of Cook’s scale, we conducted 2 statistical analyses. Firstly, we calculated the interclass correlation coefficient (ICC2) for absolute agreement (average measures) to assess the reliability of measurements made by the 3 independent evaluators. Furthermore, a 2-way Spearman’s *ρ* correlation was calculated to evaluate linear correlations among all pairs of examiners. For both tests, the strength of interrater reliability was determined according to the following criteria for statistical values: poor (<0.40), fair (0.40 to 0.59), good (0.60 to 0.74), and excellent (≥0.75) [38]. The evaluation of interrater reliability was performed on measures for week 17, for both x-ray and microCT.

The normality of the data was tested with the Kolmogorov–Smirnov test. Gaussian-distributed data were analyzed using an unpaired *t* test (2 experimental groups) or ordinary one-way analysis of variance with Tukey’s multiple comparison tests (3 or more experimental groups). Non-Gaussian-distributed data were analyzed with the Mann–Whitney *U* test (2 experimental groups) or the Kruskal–Wallis test with Dunn’s multiple comparison test (3 or more experimental groups). Data are shown as mean with standard deviation or as median with a range of minimum and maximum values. Significant *P* values are marked with asterisks: *P ≤ 0.05, **P ≤ 0.01, and ***P ≤ 0.001. The results were considered statistically significant when the probability of error (*P*) was less than 0.05. Statistical analyses were conducted using MedCalc (MedCalc Software Ltd., Belgium) and GraphPad Prism (GraphPad Software, CA, USA).

## Results

### Osteogrow-C induced rapid bridging of ulnar segmental defects

**Fig. 3.**
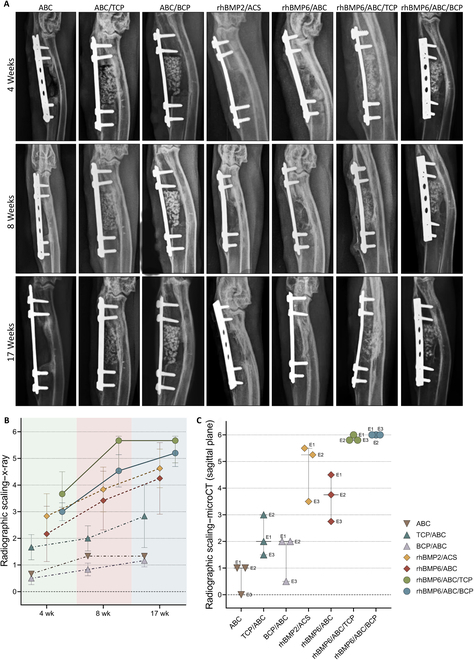
Evaluation of the healing of rabbit ulnar segmental defects. (A) In vivo images of rabbit ulnar critical size defects treated with various experimental therapies at different time points (weeks 4, 8, and 17). (B) Radiographic grading (Cook scale) of in vivo x-ray images evaluated by 3 independent examiners at weeks 4, 8, and 17. (C) Radiographic grading (Cook scale) of ex vivo micro-computed tomography (microCT) sections (sagittal plane) evaluated by 3 independent evaluators at week 17. The legend detailing the experimental groups is shown in the lower right corner.

Segmental defects were rated by using the radiographic grading scale proposed by Cook et al. [37] (Table 1) at weeks 4, 8, and 17 following the implantation of different devices in surgically created ulnar defects (Fig. 3A). At week 4 after surgery, radiographic scores were significantly higher in animals treated with rhBMP6/ABC with addition of synthetic ceramics (Osteogrow-C) than in animals treated with rhBMP6/ABC (Osteogrow) and rhBMP2/ACS (Infuse) (Fig. 3B). Specifically, Osteogrow-C implants achieved defect bridging without restoration of cortices (score 3.8 in the rhBMP6/ABC/TCP group and score 3 in the rhBMP6/ABC/BCP group). In contrast, defects treated with rhBMP6/ABC (score 2.17) and rhBMP2/ACS (score 2.83) had an increased radiodensity without defect bridging. However, defects treated with implants without BMPs (ABC, ABC/TCP, and ABC/BCP) did not change significantly compared to their postoperative appearance (scores 0.5 to 1.67).

**Table 1.** The radiographic grading scale for evaluation of segmental defects

Grade 0	No change from immediate postoperative appearance
Grade 1	Trace of radiodense material in defect
Grade 2	Flocculent radiodensity with flecks of calcification and no defect bridging
Grade 3	Defect bridged at least on one point with material of nonuniform radiodensity
Grade 4	Defect bridged on medial and lateral sides with material of uniform radiodensity, cut ends of the cortex remain visible
Grade 5	Same as grade 3, at least 1 of 4 cortices obscured by new bone
Grade 6	Defect bridged by uniform new bone, cut ends of the cortex not seen

At week 8 following implantation, cortices were significantly restored in animals treated by both Osteogrow-C formulations (score 5.27 in the rhBMP6/ABC/TCP group and 4.53 in the rhBMP6/ABC/BCP group) (Fig. 3B). On the other hand, rhBMP6/ABC and rhBMP2/ACS promoted defect bridging (scores 3.42 and 3.83, respectively) without restoration of cortices. Defects treated with ABC, ABC/TCP, and ABC/BCP did not achieve defect bridging but had an increased radiodensity compared to their postoperative appearance (scores 1.33, 2, and 0.83, respectively).

At week 17 after surgery, defects treated with Osteogrow-C formulations were bridged and their scores further increased compared to those at week 8 (score 5.4 in the rhBMP6/ABC/TCP group and 5.2 in the rhBMP6/ABC/BCP group) (Fig. 3B). Furthermore, rhBMP2/ACS and rhBMP6/ABC also achieved almost complete restoration of cortices (scores 4.63 and 4.25, respectively). Animals treated with ABC and ABC/BCP did not achieve healing of segmental defects (scores 1.33 and 1.17, respectively), while defects treated with ABC/TCP appeared to bridge at least some points on x-ray images (score 2.83). To test the interrater reliability of radiological examiners (week 17), we conducted 2 statistical tests evaluating ICC2 and rank correlation (Spearman’s *ρ*). Both tests confirmed excellent strength of reliability (Table 2).

**Table 2.** Interrater reliability measures of statistical values for ICC2 and Spearman’s *ρ* for evaluating x-ray images on week 17

Interrater reliability measure	Statistical value	CI 95% or *P* value	Strength of reliability
ICC2 (absolute agreement, average measures)	0.9028	95% CI, 0.6402–0.9662	Excellent
Spearman’s *ρ*			
Examiner 2 (N.I.) vs. examiner 1 (N.S.)	0.773	*P* < 0.0001; 95% CI, 0.512–0.903	Excellent
Examiner 3 (A.J.) vs. examiner 1 (N.S.)	0.851	*P* < 0.0001; 95% CI, 0.677–0.935	Excellent
Examiner 3 (A.J.) vs. examiner 2 (N.I.)	0.833	*P* < 0.0001; 95% CI, 0.627–0.930	Excellent

ICC2, interclass correlation coefficient; CI, confidence interval

### Osteogrow-C formulations were superior to other tested formulations in the healing of segmental defects

**Fig. 4.**
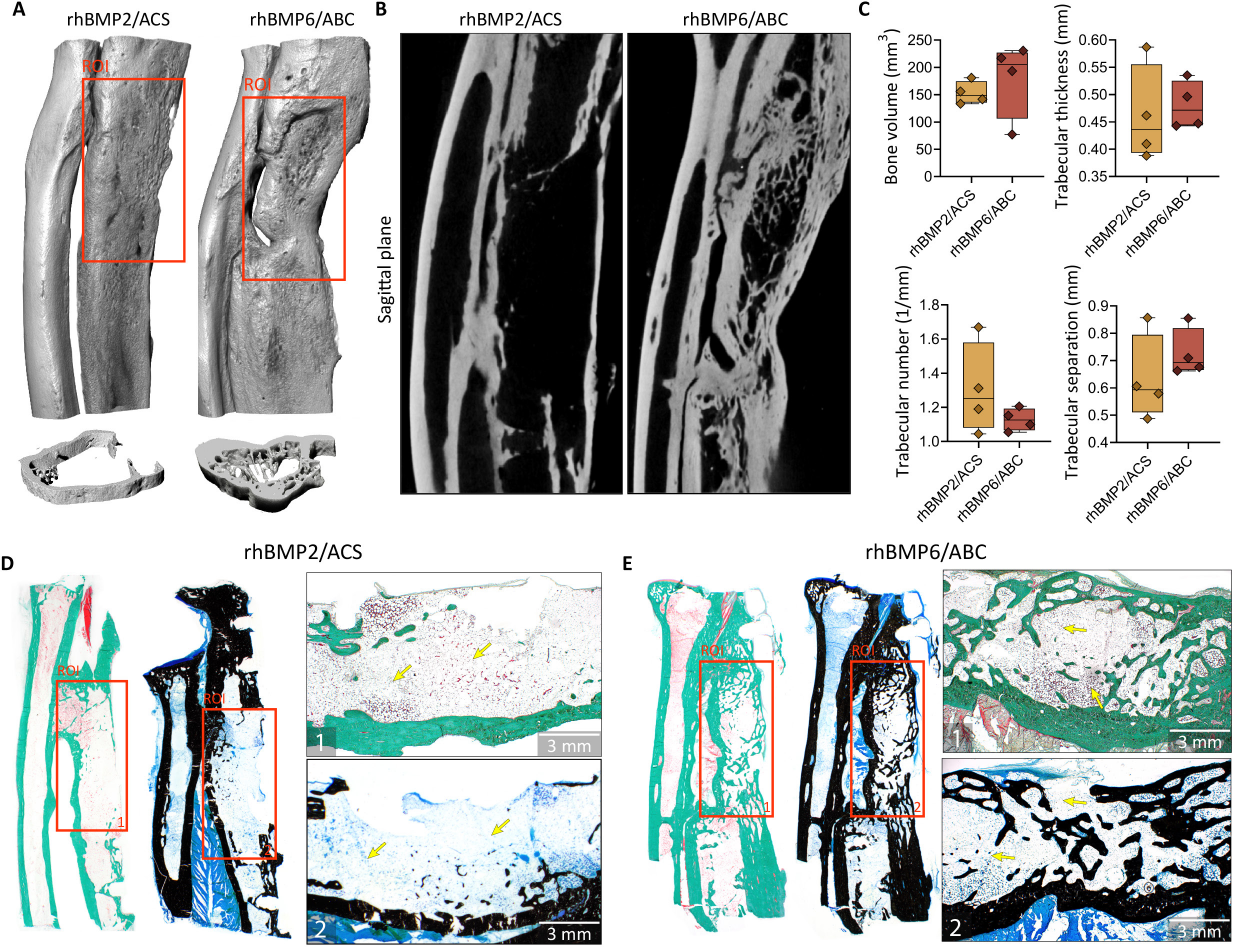
Ulnar segmental defect treated with rhBMP2/ACS (Infuse) and rhBMP6/ABC (Osteogrow). (A) Three-dimensional reconstructions and (B) microCT sections (sagittal plane) of an ulnar segmental defect treated with rhBMP2/ACS and rhBMP6/ABC. (C) MicroCT analysis of regenerated bone on the segmental defect site showing bone volume (mm^3^) and trabecular parameters including thickness (mm), number (1/mm), and separation (mm). Histological sections of whole specimens and region of interest (ROI) presenting a place of defect (orange rectangle) treated with (D) rhBMP2/ACS and (E) rhBMP6/ABC with accompanying images on higher magnifications (D and E (1 and 2)). Sections were stained by modified Goldner stain (D 1 and E 1) where the newly formed bone is green and by Von Kossa stain (D 2 and E 2) with bone in black. Yellow arrows indicate adipocytic bone marrow. Scale bars are shown in the lower right corners of D (1 and 2) and E (1 and 2).

**Fig. 5.**
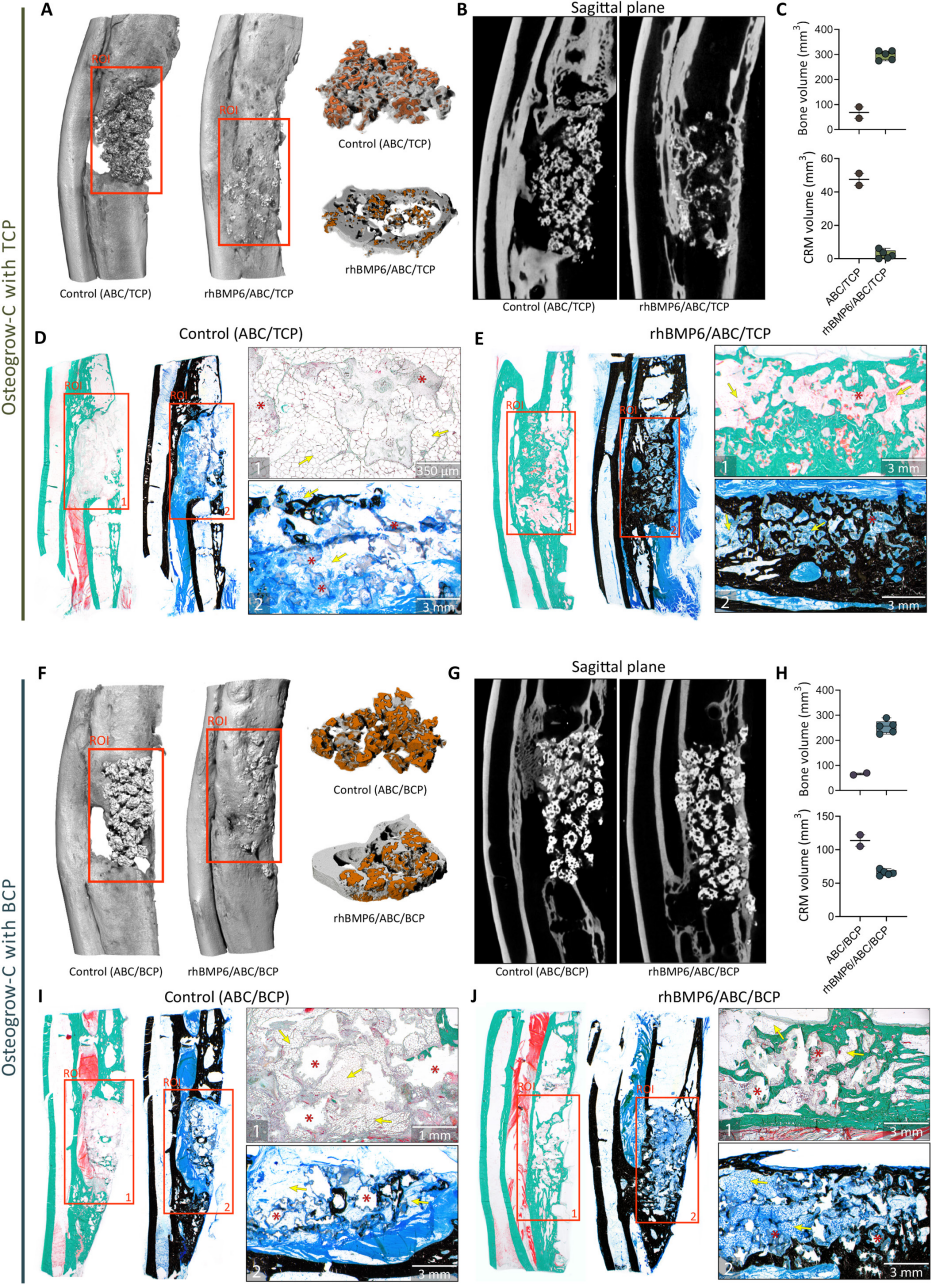
Ulnar segmental defect treated with different formulations of Osteogrow-C and appropriate controls. (A) Three-dimensional reconstructions and (B) microCT sections (sagittal plane) of ulnar segmental defects treated with Osteogrow-C containing TCP and a control group consisting of TCP in ABC. (C) Bone and CRM volume (mm^3^) of regenerated bone at the sites of segmental defects treated with control implants (ABC/TCP) and Osteogrow-C with TCP. Histological sections of whole specimens and ROI presenting a defect site (orange rectangle) treated with (D) ABC/TCP and (E) rhBMP6/ABC/TCP and with accompanying images on higher magnifications (D and E (1 and 2)). (F) Three-dimensional reconstructions and (G) microCT sections (sagittal plane) of ulnar segmental defects treated with Osteogrow-C containing BCP (rhBMP6/ABC/BCP) as CRM and control group consisting of BCP in ABC. (H) Bone and CRM volume (mm^3^) of regenerated bone at the sites of segmental defects treated with control implants (ABC/BCP) and Osteogrow-C with BCP. Histological sections of whole specimens and ROI presenting defect sites (orange rectangle) treated with (I) ABC/BCP and (J) rhBMP6/ABC/BCP and with accompanying images on higher magnifications (I and J (1 and 2)). Sections were stained by modified Goldner (D, E, I, and J (1)) and Von Kossa stains (D, E, I, and J (2)). Yellow arrows indicate adipocytic bone marrow, while asterisks present CRM. Scale bars are shown in the lower right corner.

Healing of segmental defects was further evaluated on the microCT sections (sagittal plane, with removed metal plate) obtained at the end of the experiment (week 17) using the same grading scale as for in vivo x-ray images (Figs. 3C, 4B, and 5B and G). Analyses of bone defect healing on microCT sections aligned with the findings on the x-ray (Fig. 3B). Specifically, defects were significantly healed in animals treated with rhBMP2/ACS (score 4.75) and rhBMP6/ABC (score 3.67).

Moreover, defects were completely healed in animals treated with Osteogrow-C containing TCP and BCP ceramics (scores 5.87 and 6, respectively). On the other hand, ABC (score 0.67), ABC/TCP (score 2.17), and ABC/BCP (score 1.5) induced sporadic bone formation without defect healing and restoration of cortices. The same was done for x-ray evaluation; the interrater reliability of radiological examiners was evaluated by using ICC2 and Spearman’s *ρ*. Both tests confirmed excellent strength of reliability (Table 3).

**Table 3.** Interrater reliability measures showing statistical values for ICC2 and Spearman’s *ρ* for evaluating microCT sections on week 17

Interrater reliability measure	Statistical value	CI 95% or *P* value	Strength of reliability
ICC2 (absolute agreement, average measures)	0.9313	(95% CI, 0.8248–0.9718)	Excellent
Spearman’s *ρ*			
Examiner 2 (N.I.) vs. examiner 1 (N.S.)	0.898	*P* < 0.0001; 95% CI, 0.772–0.956	Excellent
Examiner 3 (A.J.) vs. examiner 1 (N.S.)	0.855	*P* < 0.0001; 95% CI, 0.684–0.937	Excellent
Examiner 3 (A.J.) vs. examiner 2 (N.I.)	0.863	*P* < 0.0001; 95% CI, 0.700–0.941	Excellent

Histological analyses confirmed that the segmental defect was bridged in animals treated with rhBMP2/ACS, rhBMP6/ABC, and both Osteogrow-C formulations (rhBMP6/ABC/TCP and rhBMP6/ABC/BCP). Specifically, rhBMP6/ABC and rhBMP2/ACS induced restoration of cortices and medullary canal containing bone marrow (Fig. 4A to E). Since histological analyses were conducted following the removal of fixators, cortical bone was thinner in the vicinity of the fixator position (Fig. 4D and E). Defects treated with Osteogrow-C formulations completely healed by restoring cortices that formed continuity with native bone (Fig. 5A to J). Bone was present on the surfaces as well as between ceramic particles (Fig. 5A, B, E to G, and J). Bone trabeculae were surrounded with bone marrow containing both adipocytes and hematopoietic cells. On the other hand, analyses of histological and microCT sections revealed that the defect treated with TCP and BCP ceramics within ABC (without rhBMP6) was not bridged and contained only small areas of bone present between ceramic particles (Fig. 5A, B, D, F, G, and I). Moreover, newly formed bone was predominantly present in the vicinity of native bone with only sporadic areas of bone in the central portion of implants. Moreover, the residual amount of ceramics was higher than the amount of ceramics in implants containing BMP, indicating a slower resorption rate without BMPs (Fig. 5A to C and F to H).

### Synthetic ceramics increased the bone volume in BMP-based implants, while their chemical composition determined the resorption rate

**Fig. 6.**
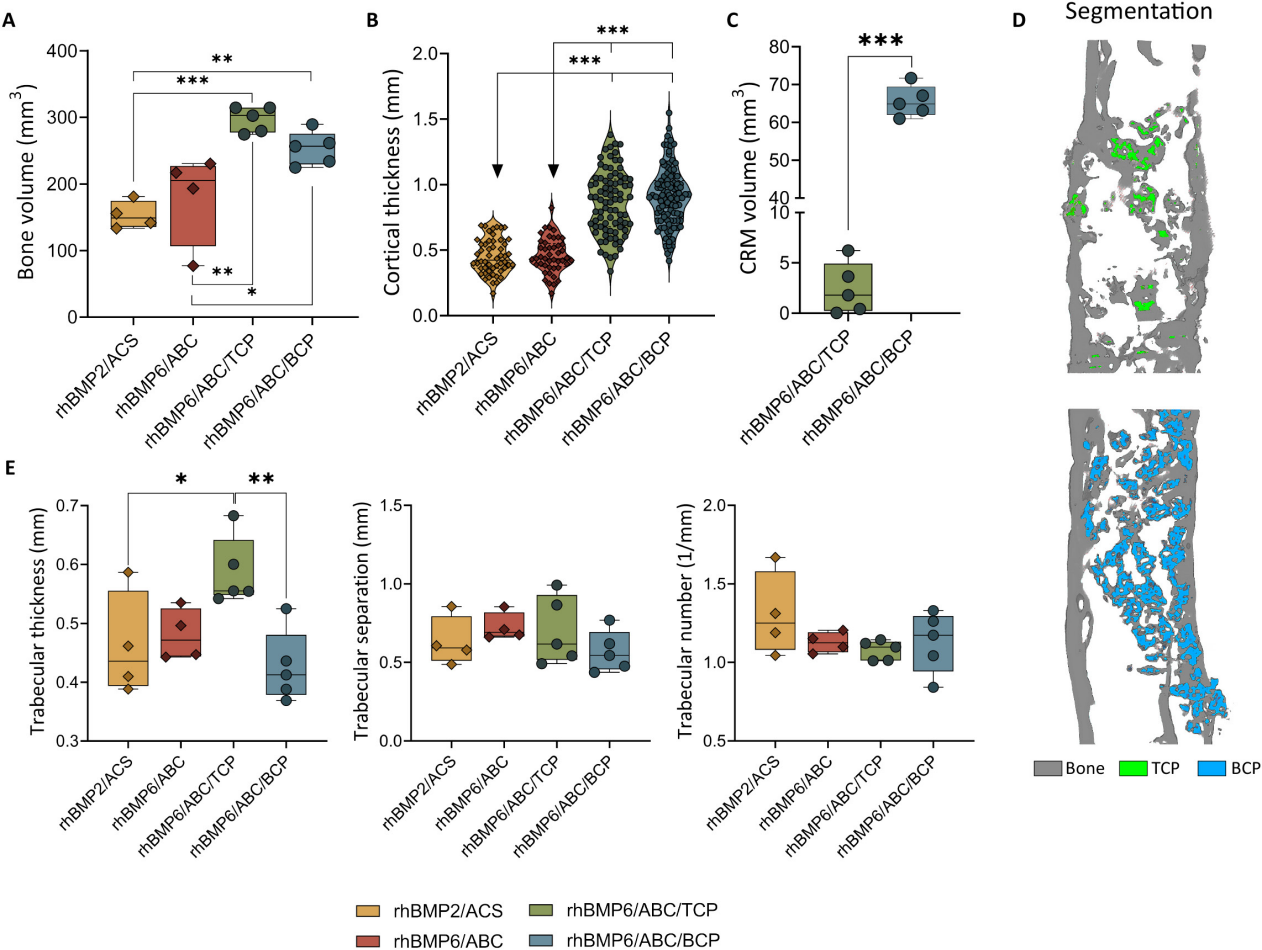
MicroCT analyses of regenerated bone 17 weeks after treatment on the sites of segmental defects treated with 4 osteoinductive formulations, namely, rhBMP2/ACS (Infuse), rhBMP6/ABC (Osteogrow), and 2 formulations of Osteogrow-C. (A) Bone volume (mm^3^) of newly formed bone at the defect site among tested formulations. (B) Measurements of cortical thickness among tested formulations. (C) Residual ceramics volume (mm^3^) among tested Osteogrow-C formulations. (D) Segmentation of ulnar bone (gray) and ceramic particles in green (TCP) and blue colors (BCP) showing the different resorption rates of ceramic particles. (E) Trabecular parameters, namely, thickness (mm), number (1/mm), and separation (mm), among experimental groups. The number of analyzed specimens was 5 or 4 per group. All *P* values below 0.05 were considered significant and are marked with asterisks: *P ≤ 0.05, **P ≤ 0.01, and ***P ≤ 0.001.

Quantification of bone volume in microCT analyses revealed that Osteogrow-C implants containing TCP or BCP induced a significantly higher amount of bone than rhBMP6/ABC as well as rhBMP2/ACS (Fig. 6A). On the other hand, bone volume was comparable between rhBMP2/ACS and rhBMP6/ABC (Figs. 4C and 6A). The primary difference among the tested formulations was cortical thickness, with Osteogrow-C formulations exhibiting a higher cortical thickness compared to both rhBMP2/ACS and rhBMP6/ABC, suggesting superior performance (Fig. 6B). Furthermore, microCT analyses of trabecular parameters showed that bone induced by Osteogrow-C with TCP ceramics had a significantly higher trabecular thickness than other experimental groups, while trabecular separation and trabecular number were comparable among all experimental groups (Fig. 6E).

MicroCT analyses showed that Osteogrow-C formulations with ceramics of different chemical compositions (TCP and BCP) significantly differed in the amount of residual ceramic particles (Fig. 6C and D). Specifically, TCP particles were almost completely resorbed, while BCP (TCP/HA with 40/60 ratio) particles were still present in significant amounts. Interestingly, microCT analyses revealed that regardless of the chemical composition of ceramics, the residual volume of ceramics was significantly lower in implants containing BMP (Osteogrow-C) than in implants containing ceramic particles within ABC (control implants without BMP) (Fig. 5C and H). Importantly, findings of microCT analyses correlated with observations in the histological sections.

## Discussion

Osteogrow-C is a novel therapeutic solution for bone regeneration composed of rhBMP6 in ABC with synthetic calcium phosphate ceramics. The safety and efficacy of different Osteogrow-C formulations were evaluated in extensive preclinical trials including rabbit and sheep PLF models [29,30,32,34,35]. Segmental defects of long bones are complex clinical conditions with limited availability of therapeutic solutions [2]. Recently, we published a case report presenting a dog with a large gunshot defect of the humerus that was treated with Osteogrow-C to avoid limb amputation [36]. Successful defect healing and restoration of limb function, in this case, demonstrated a potential for Osteogrow-C application in this indication and prompted us to evaluate it in a relevant preclinical model. In this study, we have for the first time assessed the efficacy of Osteogrow-C implants in a rabbit ulnar segmental defect model and compared it with the efficacy of implants without BMP as well as implants without synthetic ceramics.

Application of osteoinductive implants in indications where compressive forces are present requires the addition of a compression-resistant matrix to improve implant biomechanical properties [27]. Moreover, this study has demonstrated that the addition of synthetic ceramics has multiple beneficial effects on the healing of segmental defects. First, Osteogrow-C has induced accelerated defect healing as compared to rhBMP6/ABC and rhBMP2/ACS as observed on x-ray images obtained in vivo. Moreover, Osteogrow-C induced complete defect healing and the final outcome was superior compared to those of BMP-based devices without CRM as observed on x-ray images, microCT sections, microCT analyses, and histology. Previous studies on the rabbit segmental defect model have evaluated various natural and synthetic polymers, as well as inorganic materials, as carriers for BMPs [13–15,17–21]. However, due to differences in evaluation methodologies and follow-up periods, it is difficult to compare the results of different studies. Despite promising published results, none of the tested therapeutic solutions have reached high levels of technological development. On the other hand, Osteogrow and Osteogrow-C have reached a high technology readiness level and represent potential therapeutic solutions for various indications [8,9,25,26,32].

The chemical composition of ceramic particles is one of the key determinants of Osteogrow-C formulation. There is not a large number of studies comparing the impact of the chemical composition of ceramic particles on BMP-induced osteogenesis, and the research conducted so far has not provided a definitive answer regarding the optimal chemical composition of ceramics [39–41]. In our previous studies using a rat subcutaneous assay model and a rabbit PLF model, we demonstrated that ceramics with different ratios of TCP and HA can promote bone formation mediated by rhBMP6 in Osteogrow-C [29–31]. The results of this research have shown that the outcome is similar for Osteogrow-C with TCP and BCP, but analyses of in vivo radiological images have shown that TCP is superior to BCP because defect healing was achieved significantly faster. TCP is also preferred over BCP for application in segmental defects because it resorbs more quickly than BCP, thereby achieving the physiological structure of the long bone earlier. Moreover, among animals treated with ABC/ceramics, ABC/TCP achieved significantly higher radiographic scores than ABC/BCP particles, indicating that TCP possesses higher osteoconductive properties than BCP. Interestingly, our study demonstrated that regardless of the chemical compositions of the ceramic particles, the residual amount of ceramics was significantly lower in implants containing rhBMP6 (Osteogrow-C) than in control implants without BMP. This result indicates that BMPs along with induction of osteogenesis promote the resorption of calcium phosphate ceramics, which is in line with previously reported findings [42].

Recent studies suggested that ceramics alone might be sufficient to promote spinal fusion and segmental defect healing [43]. On the other hand, our study indicated that the application of calcium phosphate ceramics can promote bone formation at orthotopic sites due to its osteoconductive properties but cannot promote the healing of segmental defects during the observed period. Furthermore, the size of the segmental defect was smaller in this model compared to clinical conditions, which further reduces the likelihood that the ceramic alone can be used for the treatment of large segmental defects.

In recent years, significant efforts have been made to develop new therapeutic solutions for bone regeneration. New therapeutic strategies include the use of scaffolds seeded with mesenchymal stem cells, innovative 3-dimensional printing methods for scaffolds, and combinations of growth factors (e.g., BMP, vascular endothelial growth factor, and platelet-derived growth factor) [4,44–47]. Therapeutic strategies and drugs involving the harvesting of stem cells are considerably more complicated in terms of preparation and application and are subject to more complex regulatory frameworks than drugs that do not involve cells. On the other hand, Osteogrow-C is relatively simple to prepare, as it consists of a single osteoinductive molecule delivered in an ABC with ceramic particles. ABC is a widely available and physiologically compatible carrier of BMPs, possessing anti-inflammatory properties and allowing for the slow release of rhBMP6 after implantation [22,27]. An additional advantage of Osteogrow-C is the use of rhBMP6, which is more potent in in vitro osteoblast differentiation and in vivo bone induction compared to BMP2 and BMP7, due to its resistance to inhibition by noggin [48]. Furthermore, in our previous studies, we demonstrated that Osteogrow-C is effective with ceramic particles produced by different manufacturers, in a wide range of sizes and chemical compositions [35].

In treating large segmental defects in clinical medicine, 2 separated bone fragments are fixed using a fixator to achieve mechanical stability [49]. Although a fixator is not necessary for the rabbit ulnar segmental model, we have used a stainless steel plate as a fixator to mimic clinical conditions. Surprisingly, the Osteogrow formulation without CRM achieved slightly better outcomes in our previous study on rabbit ulnar segmental defect model without the use of a fixator [22]. Differences in the outcome might have arisen from positioning implants on the defect site after placing a metal plate, which could lead to partial disintegration of the osteoinductive implant. On the other hand, Osteogrow-C has significantly better biomechanical properties and achieved complete defect healing regardless of the implantation technique. The structural integrity and biomechanical properties of the regenerated bone were assessed by measuring the cortical bone thickness in the regenerated bone segment, as cortical bone thickness, along with cortical density, is one of the most important determinants of the biomechanical properties of the long bone diaphysis [50].

The limitations of this study include the absence of comprehensive biomechanical testing. To comply with the 4R principle (reduction, replacement, refinement, and responsibility) in the responsible conduct of animal studies, the number of samples per experimental group was defined as the minimum number that can yield reliable results according to the literature [11]. Although the selected number of samples per group was sufficient for radiological and histological analyses, we were unable to conduct biomechanical testing because it is not compatible with the histological processing of the entire sample. Moreover, the ex vivo removal of the metal fixators caused slight damage to the structural integrity of the bone, preventing us from performing biomechanical testing using direct methods, such as the 3-point bending test. Furthermore, the rabbit ulnar segmental defect model is considered an intermediate model for evaluating new regenerative therapies, and the size of the bone defect is smaller compared to typical clinical situations. For this reason, in further preclinical studies, it is necessary to conduct tests on advanced animal models that include large bone defects (e.g., the sheep tibial segmental defect model).

In conclusion, Osteogrow-C induced rapid and effective healing of ulnar segmental defects, indicating that it might be a therapeutic solution for the treatment of large bone defects in clinics. To complete the preclinical development of Osteogrow-C, future steps include testing the optimal Osteogrow-C formulation with TCP ceramics from this study on larger animals using a sheep tibial segmental defect model, leading to appropriate clinical trials.

## Data Availability

Raw data were generated at the Laboratory for Mineralized Tissues. Derived data supporting the findings of this study are available from the corresponding author (S.V.) upon request.
